# Recurrent *MDM2* Amplification in the Spectrum of *HMGA2*-Altered Pleomorphic Adenoma, Atypical Pleomorphic Adenoma and Carcinoma Ex Pleomorphic Adenoma

**DOI:** 10.1007/s12105-025-01794-y

**Published:** 2025-05-08

**Authors:** Kimberly S. T. Burghout, G. E. Breimer, S. Koppes, H. M. Hazelbag, M. L. Ooft, G. M. Raicu, A. M. Cleton-Jansen, T. van Wezel, R. van Eijk, D. Terlouw, S. L. van Egmond, V. T.H.B.M. Smit, N. J. Rupp, D. Cohen

**Affiliations:** 1 Department of Pathology, Leiden University Medical Center, Albinusdreef 2, Leiden, ZA 2333 The Netherlands; 2 Department of Pathology, University Medical Center Utrecht, Utrecht, The Netherlands; 3 Department of Pathology, Erasmus Medical Center Rotterdam, Rotterdam, The Netherlands; 4 Department of Pathology, Haaglanden Medical Center, The Hague, The Netherlands; 5 Department of Pathology, Pathology-DNA, location Rijnstate Hospital, Arnhem, The Netherlands; 6 Department of Pathology, St. Antonius Hospital, Utrecht, 3543 AZ The Netherlands; 7 Department of Otorhinolaryngology and Head and Neck Surgery, Leiden University Medical Center, Leiden, The Netherlands; 8 Department of Pathology and Molecular Pathology, University Hospital, Zürich, Switzerland; 9 University of Zürich, Zürich, 8006 Switzerland; 10 Department of Pathology, The Netherlands Cancer Institute, Antoni van Leeuwenhoek Hospital, Amsterdam, 1066 CX The Netherlands

**Keywords:** Salivary gland neoplasm, Pleomorphic adenoma, Molecular analysis, Malignant transformation

## Abstract

**Introduction:**

Pleomorphic adenoma is the most common neoplasm of the salivary glands. While the overall risk of malignancy is relatively low, a distinct molecular sub-group harboring *HMGA2* alterations seems to show an increased risk of malignant progression to carcinoma ex pleomorphic adenoma.

**Purpose:**

This study investigates *MDM2* amplification in *HMGA2*-altered pleomorphic adenoma, atypical pleomorphic adenoma, and carcinoma ex pleomorphic adenoma.

**Methods:**

In this multicenter, retrospective case series analysis, we examined 37 cases of *HMGA2*-altered pleomorphic adenoma, carcinoma ex pleomorphic adenoma, and pleomorphic adenoma with atypical features. A total of 18 cases were included from our institutional archives, with 19 additional cases derived from published literature. The cases from our institutes were analyzed for *MDM2* amplification using a stepped approach by immunohistochemistry and FISH.

**Results:**

Collectively, an *MDM2* amplification was present in 27% of pleomorphic adenoma (4 of 15), compared to 78% of carcinoma ex pleomorphic adenoma (14 of 18) (*p-value = 0.003*). In the group of pleomorphic adenomas with atypical features, an *MDM2* amplification was present in 50% of cases (2 of 4). These findings indicate an association between *MDM2* amplification and malignancy. Strikingly, a mixed control group of 12 benign and malignant *PLAG1*-altered neoplasms showed no immunohistochemical staining for MDM2.

**Conclusion:**

Immunohistochemical MDM2 expression, including *MDM2* amplification, is enriched in the group of HMGA2-altered pleomorphic adenoma, and potentially plays role in malignant progression. This study highlights the importance of recognizing the molecular sub-group of *HMGA2*-altered pleomorphic adenomas and integrate *MDM2* analysis into routine diagnostics to corroborate the cytonuclear atypia in these challenging cases.

**Supplementary Information:**

The online version contains supplementary material available at 10.1007/s12105-025-01794-y.

## Introduction

Pleomorphic adenoma (PA) and carcinoma ex pleomorphic adenoma (CXPA) are characterized by translocations involving the *HMGA2* and *PLAG1* genes [1–3]. Progression to CXPA occurs in approximately 1.5–9.5% of PA [4, 5]. However, recent findings in small case series indicate that PA harboring *HMGA2::WIF1* translocations seem to have a higher rate of regional recurrence, metastasis, and malignant transformation [6]. The observed higher rate of adverse clinical outcomes in these neoplasms calls for routine investigation and recognition by pathologists. However, most pathology laboratories do not have (unlimited) access to FISH and/or RNA-NGS analysis to identify *HMGA2* alterations. Several studies have described morphological features that could characterize *HMGA2*-altered neoplasms. Firstly, Agaimy et al. observed a distinct canalicular adenoma-like growth pattern in *HMGA2*-altered PA [7]. Though potentially helpful in identifying *HMGA2*-altered lesions, the growth pattern itself is not necessarily indicative of unfavorable biological behavior, as the described neoplasms were benign and limited follow-up data to observe local recurrence was available.

More recently, Alsugair et al. reported four (intracapsular) CXPA with *HMGA2* alterations showing striking cytonuclear atypia [8]. Besides the recurrent morphological feature of cytonuclear atypia, these lesions all showed *MDM2* amplifications. The co-amplification of *MDM2* has been described previously in salivary gland neoplasms by Persson et al., identifying *MDM2* as an amplification target in both PA and CXPA [8]. In neoplasms originating from other tissues (e.g. liposarcoma) *HMGA2* alteration and *MDM2* amplification have also been described [9, 10]. The question arises whether *MDM2* analysis could serve as a similar, widely accessible marker for identifying *HMGA2*-altered PA with unfavorable biological behavior.

In this study we aim to describe the relationship between *HMGA2*-altered lesions and *MDM2* amplification in a cohort of PA, atypical PA and CXPA, in a large case series derived from our institutional archives and published literature. Additionally, by highlighting morphological (e.g. striking cytonuclear atypia), immunohistochemical, and molecular characteristics of *HMGA2*-altered salivary gland tumors, we strive to facilitate recognition in daily diagnostic practice. To illustrate the diagnostic and clinical challenges posed by *HMGA2*-altered neoplasms neoplasms, we first present an index case involving an *HMGA2* alteration and *MDM2* co-amplification.

## Index Case

**Fig. 1:**
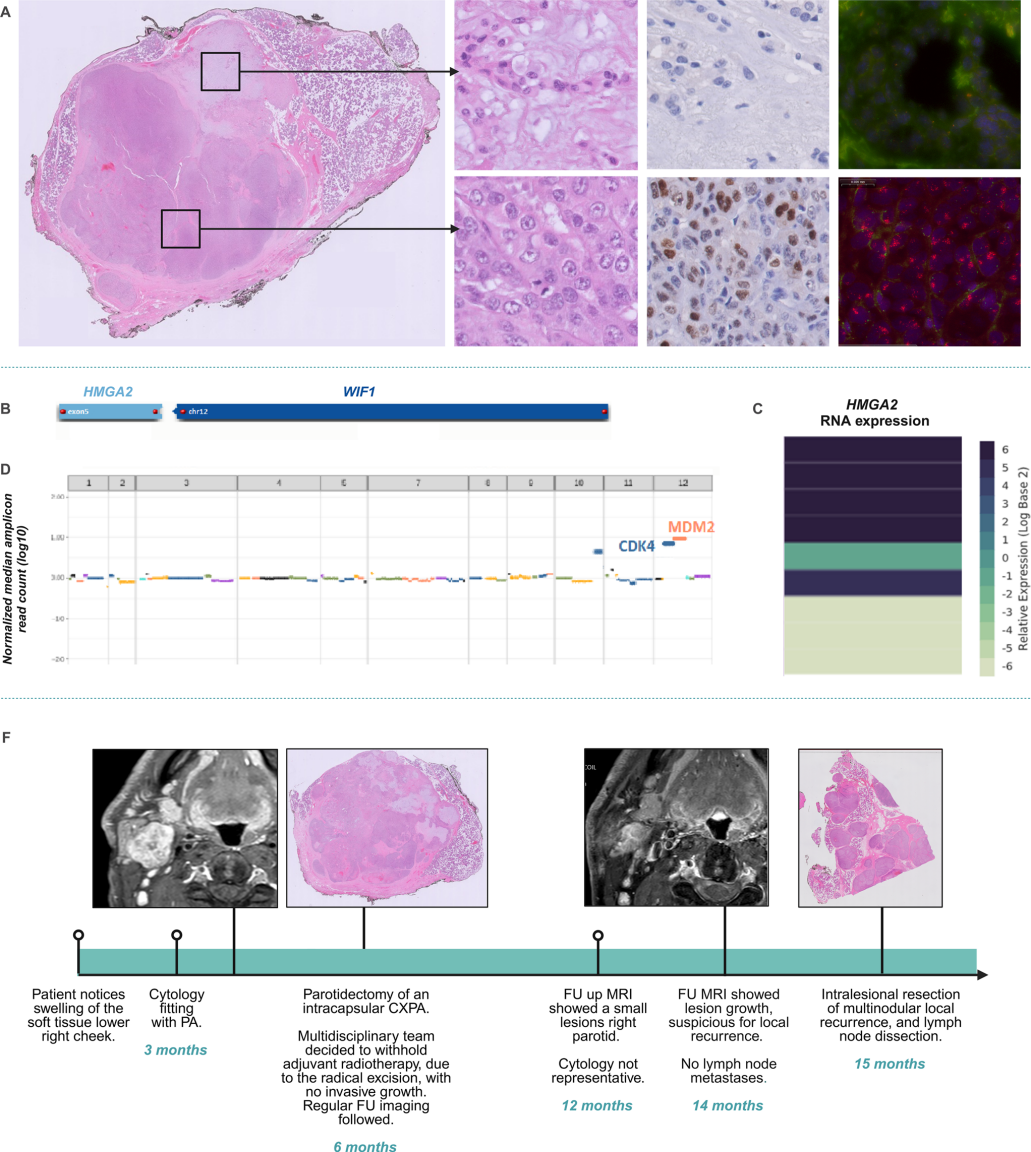
Index case. (**A**) Representative H&E slides with an overview of the lesion, and detailed images of conventional and atypical components, followed by the immunohistochemistry of MDM2 and *MDM2* FISH analysis in both components. (**B**) The *HMGA2::WIF1* fusion as analyzed by the Archer FusionPlex USZ Zurich Salivary Gland Panel v2 [11]. (**C**) The *HMGA2* gene expression is depicted in a heatmap, showing an overexpression (dark blue). (**D**) The copy number variation analysis of the atypical component showed amplification of *MDM2* and *CDK4*. (**E**) A timeline provides the details on the clinical course of the index case, highlighting the lesion’s recurrence within a year of the primary resection

A 52-year-old male presented with a lesion in the right parotid gland, reporting a minimal progression in size over time. The patient had no relevant medical history and is a never-smoker. Magnetic resonance imaging revealed a solitary, well-defined lesion of 34 mm in the deep parotid lobe. There was no clinical evidence of lymph node or distant metastases. Cytological analysis of the lesion showed a background of myxoid matrix containing grouped and individually organized cells without cytonuclear atypia, consistent with PA. Subsequent histopathological analysis of the parotidectomy specimen revealed a sharply demarcated, mostly encapsulated, multi-lobular lesion with capsular invasion and two morphological components. Firstly, the hypocellular component of the lesion consisted of chondromyxoid stroma, uniform spindle cells, and uniform epithelial cells arranged in fields and glandular structures. Secondly, the predominant component of the lesion consisted of a hypercellular population of epithelial cells with enlarged, irregularly shaped nuclei with prominent nucleoli and substantial eosinophilic cytoplasm, in a background of thick hyalinized stroma. In this component, there was increased mitotic activity, including atypical mitoses, and areas of necrosis. Immunohistochemical analysis confirms the biphasic architecture of the lesion, with the epithelial cells expressing keratin AE1/AE3 and keratin 7 and the myoepithelial cells expressing S100, calponin and p63. In the hypercellular component of the lesion, a more variable staining pattern was observed with a partially retained biphasic immunohistochemical pattern and areas of myoepithelial differentiation. Ki-67 showed a proliferation rate of 1–2% in the conventional component and an increased proliferation rate of up to 20% in the hypercellular component, showing striking cytonuclear atypia. No invasive growth in surrounding salivary gland tissue was observed, and no perineural or vascular invasion was found. Molecular analysis was performed using fusion-analysis (Archer FusionPlex USZ Zurich Salivary Gland Panel v2 [11]), detecting the same *HMGA2*(exon 5)::*WIF1*(intron 2) fusion in both the conventional and atypical component. The re-arrangement involved a full-length *HMGA2* gene, fused to an inversed intronic sequence of *WIF1*, resulting in an overexpression of the *HMGA2* gene. Next Generation Sequencing (NGS AmpliSeq LUMC Cancer Hotspot Panel version 6) of the isolated DNA of the atypical component showed no pathogenic variations (e.g. *TP53* mutation) but copy number variation analysis revealed the presence of *MDM2* and *CDK4* co-amplification. The *MDM2* amplification in the atypical component was visualized using immunohistochemistry, showing no staining in the conventional component and strong nuclear expression in the atypical component. Amplification was confirmed by *MDM2* FISH analysis. Based on the prominent atypical component with increased mitotic activity, the multi-lobular growth and the molecular findings, the lesion was classified as an intracapsular CXPA. Eleven months after the complete resection with a 1 mm margin, the patient developed multinodular local disease recurrence that was surgically resected with positive margins. The case, including the timeline, is highlighted in Fig. 1.

## Materials and Methods

### Literature Case Selection

The literature study followed the PRISMA guideline for systematic reviews [12]. An inquiry of the PubMed database was performed using search terms related to “pleomorphic adenoma”, “carcinoma ex pleomorphic adenoma”, “salivary gland”, “*HMGA2*” and “*MDM2*”. Abstracts of the retrieved articles were screened and selected for full-text review when describing cases of adults with *HMGA2* rearranged salivary gland neoplasms. For the final selection, the following exclusion criteria were used: (1) lack of essential clinical and pathological details (e.g. histopathological diagnosis), (2) outdated molecular analysis (e.g. karyotyping), and (3) metastases of carcinoma ex pleomorphic adenoma. Cases published multiple times were included only once in the case series. Eligible study designs for this literature analysis included case-control series, case reports, cohort studies, and cross-sectional studies. A database was composed of the *HMGA2*-rearranged, *MDM2*-analyzed PA and CXPA described in literature, detailing patient sex, age, and histopathological diagnosis. When available the details on tumor location, size, morphology, and follow-up were also collected. The histological diagnosis of literature-derived cases was not reviewed due to limited availability histological images.

### Case Series Analysis

Medical ethical approval was granted by the ethics committee Leiden-Den Haag-Delft. Due to the retrospective design of the study, informed consent was waived. However, written informed consent was obtained from the patient described in the index case. Clinical details, including age, sex, lesion size and location, and molecular characteristics, were collected. All specimens were routinely processed using formalin-fixation and paraffin embedding (FFPE) for histopathological and molecular analysis, and data was collected retrospectively. In addition to the index case, PA, atypical PA and CXPA with known *HMGA2* alterations were retrospectively selected and included from six hospitals (Leids Universitair Medisch Centrum, Universitair Medisch Centrum Utrecht, Erasmus Medisch Centrum Rotterdam, Rijnstate Ziekenhuis Arnhem, St. Antonius Ziekenhuis Utrecht and Gelre Ziekenhuis Apeldoorn). The final diagnosis was made according to the definitions of the WHO Head and Neck Tumours classification (5th edition). Distinguishing between an atypical PA and an intracapsular CXPA remains a challenge, as a universally accepted diagnostic definition has yet to be established. The literature describes several histomorphological, immunohistochemical and molecular features of the often cellular, non-invasive lesions with potentially different biological behavior [13–15]. Based on these descriptions the following definitions were applied in this study: atypical PA were defined as lesions showing significant cytonuclear atypia, not reaching the extend of cytonuclear atypia observed in CXPA, with preserved architecture of layered ductal and myoepithelial components. Additionally, lesions showed no worrisome immunohistochemical or molecular features such as p53 mutation or HER2 amplification. Lesions with significant cytonuclear atypia, increased mitotic activity (typically > 2 per 10HPF, abnormal mitotic figures, high proliferation index in ki67), areas of disturbed architecture and/or presence of a distinct malignant component, were classified as intracapsular CXPA. These lesions were reviewed independently by two experienced Head and Neck pathologists (DC, NJR, GEB),

### Immunohistochemistry

Immunohistochemical analysis for MDM2 was performed on 3-µm thick FFPE sections, automatically stained with the MDM2 monoclonal IF2 antibody clone on the Dako Omins stainer in Leiden University Medical Center. The remaining cases were analyzed by immunohistochemical staining for MDM2 (Invitrogen, clone IF-2), conducted using a pretreatment protocol involving 24 min of CC1 buffer, followed by a 32-minute incubation with the primary antibody and staining carried out on the Ventana Benchmark Ultra platform. Two observers reviewed the MDM2 staining (KSTB and DC). When possible, all cases were additionally analyzed for *MDM2* amplification using *MDM2* FISH or NGS Cancer Hotspot analysis. As a control group, 12 cases of PA, atypical PA and CXPA with known *PLAG1* fusion were also analyzed using MDM2 immunohistochemistry, followed by FISH analysis if any amount of nuclear staining was present.

### FISH Analysis

In Leiden, the *MDM2* FISH was performed on FFPE sections of 4µM, processed using the Vysis *MDM2*/CEP12 FISH probe to confirm *MDM2* amplification, and FISH was performed using the Histology Fish Accessory Kit (Dako, Glostrup, Denmark) as per the manufacturer’s description. Nuclei were counterstained with 10 µl of 4′,6-diamidino-2-phenylindole (DAPI). The FISH for *MDM2* and *HMGA2* performed elsewhere used the Zytovision probe kit (Zytovision.GmbH, Bremerhaven, Germany), as per the manufacturer’s description.

### Molecular Analysis

*HMGA2* alterations were detected using RNA sequencing. RNA was extracted from FFPE blocks, and samples were analyzed using the SalvGlandDx RNA Fusion Panel on the Illumina MiSeq system [11]. Additional analysis of *MDM2* amplification was performed on DNA isolates using the in-house developed NGS Ampliseq Cancer Hotspot Panel on the Genexus Ion Torrent S5 platform (Thermo Fisher Scientific, Canada).

### Statistical Analysis

Statistical analysis was performed using IBM SPSS software (version 29). The PA-group and CXPA-group were compared on *MDM2* status using the Fishers’ exact test. The atypical PA were excluded from the analysis due to the uncertain biological behavior and small sample size. Statistical significance was set at a *p-value* of < 0.05. To assess the strength of the association between *MDM2* amplification and malignant histopathological diagnoses in the *HMGA2*-altered lesions, the odds ratio (OR) was calculated. For this, PA with atypical features were included in the benign category. Additionally, a logistic regression analysis was performed to assess whether histological diagnosis (benign/malignant) and *MDM2* amplification status were associated with the occurrence of a clinical event (e.g. local recurrence, metastasis, malignant transformation). For the logistic regression and OR analysis, PA with atypical features were included in the benign category.

## Results

### Literature Cases

From the literature search a total of five studies detailing 19 cases of *HMGA2*-altered, *MDM2-*analyzed salivary gland neoplasms were included [6, 8, 16–18]. The studies were published between 1998 and 2024. Various molecular investigations were used, including NGS analysis, RNA sequencing, *MDM2* FISH analysis, array-comparative genomic hybridization (aCGH), and Southern blot analysis. Further case-specific characteristics are described in the next Sect. (4.2 case series analysis).

### Case Series Analysis

#### Clinical and Demographic Features

Retrospectively an additional 18 cases with *HMGA2* alteration were identified from six different hospitals. Combining the cases derived from literature and the additional cases from our clinical practice, a total of 37 *HMGA2*-altered salivary gland neoplasms analyzed for *MDM2* amplifications were collected for this analysis. The cohort included 24 female patients (65%) and 13 male patients (35%), with ages ranging from 25 to 86 years old (mean 62 years) at the time of diagnosis. A total of 31 lesions derived from the parotid gland (84%), 2 from the parapharyngeal space (5%), one from the hard palate (3%), one from the mandibular tonsil lodge (3%), and one of unknown origin (3%). The lesion size varied between 7 and 53 mm (mean 25 mm). Among the cohort, there were 15 pleomorphic adenomas, 4 pleomorphic adenomas with atypical features, 1 intracapsular carcinoma ex pleomorphic adenoma, 1 carcinoma ex pleomorphic adenoma with minimal invasion, and 16 carcinoma ex pleomorphic adenoma. An overview of patient and lesion characteristics can be found in Table 1.

**Table 1:** Characteristics of 37 *HMGA2*-altered cases from literature and clinical practice with investigated *MDM2* status

Case	Age	Gender	Location	Histopathological diagnosis	Subtype / growth pattern	HMGA2 alteration	HMGA2 analysis	MDM2 amplification	MDM2 analysis
A	52	M	Parotid gland	CXPA intracapsular	epithelial-myoepithelial carcinoma	*HMGA2::WIF1*	RNA-NGS	Yes	FISH
B	32	F	Parotid gland	PA atypical features	canalicular-like with solid areas	*HMGA2::WIF1*	RNA-NGS	Yes	FISH
C	75	M	Mandibula tonsil	PA	solid	*HMGA2::WIF1*	RNA-NGS	No	FISH
D	80	M	Parotid gland	PA	canalicular-like	*HMGA2::RAP1B*	RNA-NGS	No	DNA-NGS
E	77	M	Parotid gland	PA	canalicular-like	*HMGA2::WIF1*	RNA-NGS	No	DNA-NGS
F	71	M	Parotid gland	PA	trabecular	*HMGA2::WIF1*	RNA-NGS	No	FISH
G	42	M	Parotid gland	PA	canalicular-like with conventional component	*HMGA2::WIF1*	RNA-NGS	No	FISH
H	68	F	Parapharyngeal	PA	canalicular-like with conventional and solid component	*HMGA2* loss	FISH	No	FISH
I	53	F	Parotid gland	PA	canalicular-like	*HMGA2* ampl.	FISH	No	FISH
J	76	F	Parotid gland	CXPA minimal invasion	salivary duct carcinoma	*HMGA2* ampl.	FISH	No	DNA-NGS
K	68	F	Parapharyngeal	PA	canalicular-like with conventional component	*HMGA2* ampl.	FISH	No	FISH
L	72	M	Parotid gland	PA	canalicular-like	*HMGA2* ampl.	FISH	No	FISH
M	76	F	Palatum durum	PA atypical features	canalicular-like	*HMGA2* ampl.	FISH	Yes	FISH
N	80	M	Parotid gland	PA	canalicular-like	*HMGA2* RNA overexpression	RNA-NGS	No	IHC
O	54	F	Parotid gland	PA atypical features	canalicular-like with solid component and sclerosis	*HMGA2::WIF1*	RNA-NGS	No (single cells)	FISH
P	70	M	Parotid gland	CXPA	salivary duct carcinoma	*HMGA2::FLJ41278*	RNA-NGS	No (single cells)	FISH
Q	41	F	Parotid gland	PA	conventional with sclerosis	*HMGA2::WIF1*	RNA-NGS	No	IHC
R	69	M	Parotid gland	PA atypical features	in situ salivary duct carcinoma	*HMGA2::FLJ41278*	RNA-NGS	No (single cells)	FISH
1	25	*F*	*NDA*	CXPA	*NDA*	*HMGA2* ampl./gain	aCGH	Yes	FISH
2	55	F	Parotid gland	PA	*NDA*	*HMGA2* ampl./gain	FISH	Yes	FISH
3	85	F	Parotid gland	PA	*NDA*	*HMGA2* ampl./gain	FISH	Yes	FISH
4	86	F	Parotid gland	CXPA	*NDA*	*HMGA2::WIF1*	RT-PCR	No	FISH
5	76	M	Parotid gland	CXPA	*NDA*	*HMGA2::WIF1*	RT-PCR	Yes	FISH
6	74	F	Parotid gland	PA	*NDA*	*HMGA2::WIF1*	RT-PCR	Yes	FISH
7	37	F	Parotid gland	PA	*NDA*	*HMGA2::WIF1*	aCHG	Yes	FISH
8	73	M	Parotid gland	CXPA	*NDA*	*HMGA2::WIF1*	RT-PCR	No	FISH
9	35	F	Parotid gland	CXPA	*NDA*	*HMGA2* ampl./gain	FISH	Yes	FISH
10	40	F	Parotid gland	CXPA	*NDA*	*HMGA2* ampl./gain	FISH	Yes	FISH
11	70	F	Parotid gland	CXPA	*NDA*	*HMGA2* ampl./gain	FISH	Yes	FISH
12	69	M	Parotid gland	CXPA	*NDA*	*HMGA2* ampl./gain	FISH	Yes	FISH
13	66	F	Parotid gland	CXPA	*NDA*	*HMGA2* ampl.	DNA-NGS	Yes	DNA-NGS
14	30	F	Parotid gland	CXPA minimal invasion	*NDA*	*HMGA2* ampl.	DNA-NGS	Yes	DNA-NGS
15	32	F	Parotid gland	CXPA	hypercellular and monomorphic	*HMGA2::GRIP1*	RNA-NGS	Yes	FISH
16	51	F	Parotid gland	CXPA	hypercellular and eosinophilic	*HMGA2::PLXNC1*	RNA-NGS	Yes	FISH
17	89	F	Parotid gland	CXPA	conventional with infiltration	*HMGA2* ampl.	IHC	Yes	FISH
18	67	F	Parotid gland	CXPA	hybrid with infiltration	*HMGA2* ampl.	IHC	Yes	FISH
19	60	F	Buccal mucosa	CXPA	basaloid features	*HMGA2::WIF1*	RNA-NGS	Yes	DNA-NGS

PA = pleomorphic adenoma, CXPA = carcinoma ex pleomorphic adenoma, NDA = no data available, NGS = Next Generation Sequencing, FISH = Fluorescence In Situ Hybridization, aCGH = arary *Comparative Genomic Hybridization*,* PCR = Polymerase Chain Reaction*

Follow-up and survival data were collected when available, with FU ranging between 5 and 854 months in PA, 1-252 months in PA with atypical features and 4-192 months in CXPA [6, 8, 16]. Adverse outcomes (local recurrence, metastases, death due to the disease) were reported in 4 of 37 cases. Case A (CXPA with *MDM2* amplification) showed local recurrence 11 months after the primary radical resection. Case C (PA without *MDM2* amplification) showed LR after a primary R1 resection. Case 8 (CXPA without *MDM2* amplification) showed LR. Case 19 (CXPA with *MDM2* amplification) showed LR after 5 months, distant metastasis after 31 months, and death due to the disease after 73 months. A complete overview of follow-up and survival data can be found in Table 2.

### Pathological Features

Within the cohort there were 15 PA, 4 PA with atypical features, and 18 CXPA. The PA showed morphological variety with conventional chondromyxoid stroma, with varying cellular solid growth, trabecular and canalicular-like growth. Immunohistochemically, the lesions showed a bilayered architecture, with an inner epithelial cell layer and outer myoepithelial cell layer. In one case (case Q), cytonuclear atypia was seen in a background of extensive regressive changes, without other malignant features. The histological type of the malignant component of CXPA included epithelial-myoepithelial carcinoma and salivary duct carcinoma. Four PA with atypical features were included, showing some cytonuclear atypia and uncertain invasive growth, insufficient for a diagnosis of CXPA. In case R, a small 7 mm lesion showed invasive growth and in situ salivary duct carcinoma. An overview of the H&E, histological sub-type/growth pattern, MDM2 staining pattern, *MDM2* amplification, and molecular alteration of cases A-R can be found in Supplementary Fig. 1. In the *PLAG1*-altered control group of 12 PA, PA with atypia and CXPA there was no immunohistochemical staining of MDM2, as can be seen in Supplementary Fig. 2.

**Table 2:** Follow-up and survival data

Case	Histopathological diagnosis	MDM2 amplification	Follow-up (months)	Survival	Details
A	CXPA intracapsular	Yes	13	LR	LR (multi-nodular) at 11 months
B	PA atypical features	Yes	252	NED	-
C	PA	No	200	LR	LR (multi-nodular) 2x at 177 and 190 months
D	PA	No	10	NED	-
E	PA	No	11	NED	-
F	PA	No	6	NED	-
G	PA	No	30	NED	-
H	PA	No	18	DOC	Liver cancer
I	PA	No	*NDA*	*NDA*	*NDA*
J	CXPA minimal invasion	No	13	NED	-
K	PA	No	19	NED	-
L	PA	No	26	NED	-
M	PA atypical features	Yes	20	NED	-
N	PA	No	12	NED	-
O	PA atypical features	No	10	NED	-
P	CXPA	No	30	NED	-
Q	PA	No	5	NED	-
R	PA atypical features	No	1	NED	-
1	CXPA	Yes	*NDA*	*NDA*	*NDA*
2	PA	Yes	204	NED	-
3	PA	Yes	854	DOC	-
4	CXPA	No	60	NED	-
5	CXPA	Yes	36	DOC	-
6	PA	Yes	36	NED	-
7	PA	Yes	36	NED	-
8	CXPA	No	84	LR	Local recurrence
9	CXPA	Yes	156	NED	-
10	CXPA	Yes	72	NED	-
11	CXPA	Yes	192	NED	-
12	CXPA	Yes	96	DOC	-
13	CXPA	Yes	*NDA*	*NDA*	*NDA*
14	CXPA	Yes	*NDA*	*NDA*	*NDA*
15	CXPA	Yes	5	NED	-
16	CXPA	Yes	4	NED	-
17	CXPA	Yes	5	NED	-
18	CXPA	Yes	4	NED	-
19	CXPA	Yes	73	DOD	LR 5 months, metastases 31 months, DOD 73 months

PA = pleomorphic adenoma, CXPA = carcinoma ex pleomorphic adenoma, NDA = no data available, NED = no evidence of disease, LR = local recurrence, DOC = died of other causes, DOD = died of disease, AWDM = alive with distant metastases

### Molecular Analysis

Molecular analysis showed 19 cases with a confirmed *HMGA2* rearrangement, paired with a variety of fusion partners, including *WIF1* (*n* = 14), *RAP1B* (*n* = 1), *FLJ41278* (*n* = 2), *GRIP1* (*n* = 1), and *PLXNC1* (*n* = 1). Case N showed increased RNA expression of *HMGA2* without a confirmed fusion. Two cases showed immunohistochemical overexpression of HMGA2 without a confirmed fusion [16]. Six cases showed a break apart signal of *HMGA2* in FISH analysis. The remaining nine cases showed amplification or gain of *HMGA2*.

**Fig. 2:**
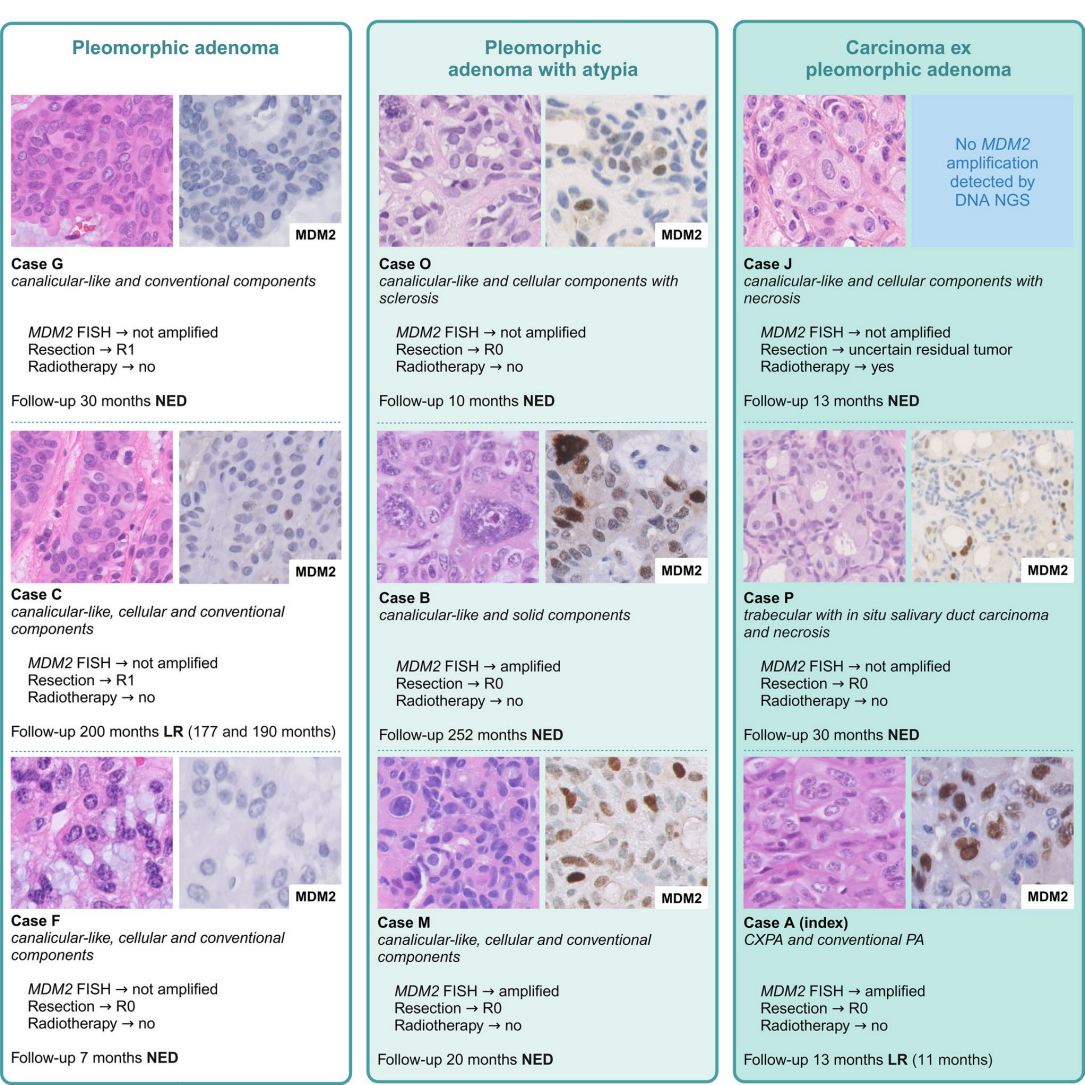
An overview of nine cases, showing the morphological spectrum of benign, atypical and malignant *HMGA2*-altered cases analyzed for *MDM2* amplification and providing additional data on the residual tumor classification, radiotherapy, follow-up and survival. Additional high power images of all nine cases can be found in Supplementary Fig. 3

*MDM2* amplification was present in 20 of 37 cases (54%); respectively in 4 of 15 PA (27%), in 2 of 4 PA with atypical features (50%), and in 14 of 18 CXPA (78%) (*p-value 0.003*). The odds ratio OR was 7.6, indicating a strong association between a malignant histopathological diagnosis and the presence of *MDM2* amplification. However, further logistic regression analysis showed a non-significant association between clinical outcome event and *MDM2* amplification (OR = 2.16; 95% CI, 0.20-22.96; *p-value* 0.522), and a non-significant association between clinical outcome event and malignant classification (OR = 0.17; 95% CI, 0.01–2.31; *p-value 0.183*).

**Fig. 3:**
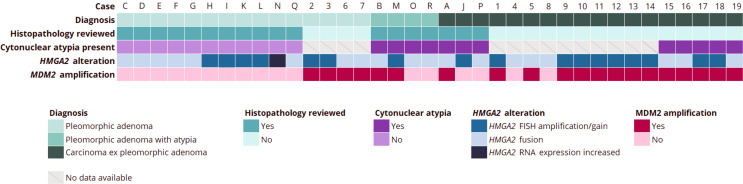
A schematic overview of all *n* = 37 cases included in the case series anlaysis, detailing the final histopathological diagnosis, the presence of cytonuclear atypia, the detected *HMGA2* alteration and the presence of the *MDM2* amplification. The histopathological diagnosis of cases derived from the literature was not reviewed

Example cases of benign, atypical, and malignant cases with the MDM2 immunohistochemical staining pattern and clinical data can be found in Fig. 2. Three cases (case O, P and R) showed MDM2 immunohistochemical positivity of single atypical cells, in which *MDM2* amplification could not be confirmed by FISH analysis. Similar to the index case, some cases in literature showed amplification of both *MDM2* and *CDK4*. A schematic representation of all cases can be found in Fig. 3.

## Discussion

This retrospective, multicenter case series analysis combines 18 cases from our clinical practice with an additional 19 cases from the literature, providing a cohort of 37 benign, intermediate, and malignant *HMGA2*-altered salivary gland neoplasms analyzed for *MDM2*. We found an *MDM2* amplification in 27% of the PA, as opposed to 78% of the CXPA (*p-value 0.003*). This finding highlights the correlation between *HMGA2* alteration and *MDM2* amplification in salivary gland neoplasms, as has been described in other neoplasms (e.g. well-differentiated and dedifferentiated liposarcoma, rhabdomyosarcoma, lipoma, uterine leiomyoma, spindle cell sarcoma) [19–23].

Due to the enriched presence of *MDM2* amplification in CXPA with *HMGA2* alteration, recognition of this molecular sub-group of salivary gland neoplasms could be potentially informative in the pathologists’ assessment. Previous studies have indicated potential morphological clues like the presence of cytonuclear atypia and a canalicular-like growth pattern [7, 16]. In the current analysis, PA with a *HMGA2* fusion often showed a cellular morphology, sometimes with canalicular, trabecular and/or conventional PA growth pattern (Supplementary Fig. 1). Notably, canalicular-like architecture was a common but not an exclusive morphological feature in this case series (canalicular-like growth in 11 of 18 *HMGA2*-altered lesions versus 1 of 12 *PLAG1*-altered lesions) and should therefore be used with care when identifying *HMGA2*-altered lesions.

Cytonuclear atypia, described by Alsugair et al. (2024) in CXPA was a more reproducible feature in this analysis, potentially indicating an underlying *MDM2* amplification [16]. Interestingly, in the *PLAG1* control group, a mix of both benign, intermediate, and malignant lesions, no immunohistochemical positivity for MDM2 was found, despite the occasional presence of cytonuclear atypia (Supplementary Fig. 2). This suggests that MDM2 immunohistochemical positivity may be a potential diagnostic feature for identifying the underlying *HMGA2* alterations in PA and CXPA. However, when suspected, *MDM2* amplification should be evaluated using FISH. Like analysis of Human Epidermal growth factor Receptor 2 (HER2), Androgen Receptor (AR) and p53 staining, *MDM2* analysis could be added to the pathologists’ toolbox for difficult to classify salivary gland neoplasms, as a surrogate for the underlying molecular alteration [24–26]. Although *MDM2* amplification was more frequently observed in *HMGA2*-altered PA with atypia and CXPA, our clinical outcome data, albeit limited, do not show a different clinical outcome in these tumors. With exception of the index case, in most cases, including in the *MDM2*-amplified tumors, no local recurrence or other adverse clinical outcome was observed. Therefore it is important to note that while *MDM2* analysis may serve as a helpful tool in explaining cytonuclear atypia, it should not be interpreted as a definitive marker of malignancy or poor prognosis at this point. Conversely, a lack of *MDM2* amplification may further reassure the pathologist when they are faced with a diagnostically challenging case, as it supports a benign classification.

In this analysis the consequence for PA with atypical features and the relevance of *MDM2* amplification remains unclear. We included four PA with striking atypical features (case B, M, O and R) lacking invasive growth after extensive tissue sampling. This could be interpreted as lesions potentially transitioning from benign to malignant neoplasms. Case B and M showed cytonuclear atypia and *MDM2* amplification, with no evidence of disease after extensive follow-up periods of respectively 252 and 20 months. Case O and R, showed cytonuclear atypia with staining of single, atypical cells in MDM2, with no proven *MDM2* amplification and no evidence of disease after respectively 10 months and 1 month. However, immunohistochemical expression could certainly be indicative of otherwise altered signaling pathways independent of amplification [27]. The limited number of cases did not allow further interpretation of the implication of *MDM2* amplification in the atypical but not overt malignant lesions. Strikingly, however, nuclear staining of single, atypical cells was observerd in case O and Case R. In the *PLAG1* control group no staining of *MDM2* staining of single atypical cells was encountered, emphasizing the connection between *HMGA2* alteration and *MDM2* amplification in PA. For daily practise, this implies, that despite atypical features and MDM2 expression and/or amplification, the growth pattern and cytology still remain the primary diagnostic criteria of CXPA.

**Fig. 4:**
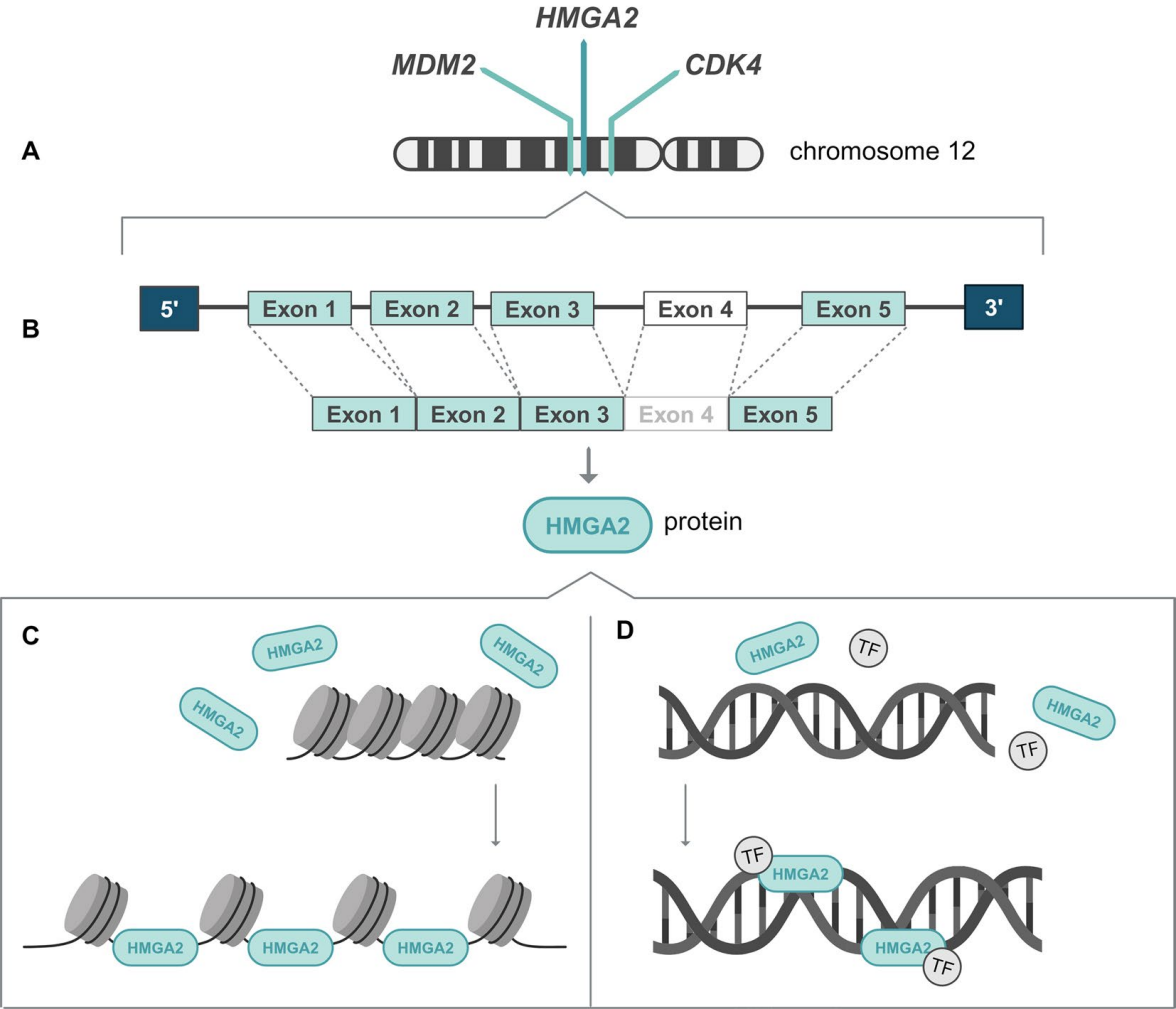
A schematic overview of *HMGA2* gene expression. (**A**) The *HMGA2*, *MDM2* and *CDK4* genes are located on chromosome 12. (**B**) The *HMGA2* gene consists of five exons, encoding the HMGA2 protein. (**C**) The HMGA2 protein binds to AT-rich domains of DNA and influences gene expression of the DNA by altering chromatin compactness, (**D**) and by facilitating the binding of transcription factors to the acidic C-terminal tail of the HMGA2 protein and the DNA

The exact relationship between *MDM2* and *HMGA2* alterations in salivary gland neoplasms remains unclear, however, we propose a potential mechanism. The *HMGA2* gene is located on the long arm of chromosome 12 and consists of five exons, of which the first three exons encode for an adenine-thymine (AT)-hook binding domain that enables the HMGA2 protein to interact with the minor grooves of the DNA. Physiologically the gene is expressed during early life, with a role in DNA repair by facilitating the binding of transcription factors and altering the chromatin structure (Fig. 4) [28]. Rearrangement of *HMGA2* is suggested to drive tumorigenesis by the (dis-)regulatory effect of the HMGA2 protein on *PLAG1* and *IGF2* expression, a suggestion based on the downregulation of *IGF2* expression in patients with Silver-Russel syndrome. This syndrome involves mutations in *HMGA2* or the *HMGA2* regulated gene *PLAG1* [3]. The observed coamplification of *MDM2* in *HMGA2*-rearranged PA and CXPA may result from the ability of the HMGA2 protein to alter chromatin structure, thereby increasing chromosomal instability with subsequent DNA fragmentation, inaccurate repair, and gene amplifications located downstream of *HMGA2* on chromosome 12 [29–31]. Further molecular studies and cell biological studies might unravel this complex relationship.

To create a larger case series and explore the correlation between molecular alteration and clinical outcome, cases from previous literature and clinical practice were combined. A key limitation is the lack of morphological review of the literature-derived cases, including four lesions classified as PA with *MDM2* amplification. This precluded the assessment of atypia/atypical features, potentially overestimating the occurrence of an *MDM2* amplification in *HMGA2*-altered PA. Furthermore, RNA fusion analysis of cases derived from our institutional archives was mostly performed when canalicular-like/cellular morphology, cytonuclear atypia, or invasive growth were encountered. This selective approach introduced bias, resulting in 42% CXPA, a high percentage compared to the overall risk of malignant transformation in PA, which is reported to be between 1.5 and 9.5%, and up to 20% in *HMGA2::WIF1* altered cases [2, 4, 5]. Due to the current limited use of molecular analysis of PA and CXPA, a high-quality, prospective cohort of *HMGA2*-altered neoplasms is difficult to collect. This is a common limitation in salivary gland neoplasm research, partly owing to the rarity of (malignant) tumors, underscoring the need for close collaborations in the future.

In conclusion, we found that immunohistochemical MDM2 expression and *MDM2* amplifications are frequent in *HMGA2*-altered salivary gland tumors with cytonuclear atypia, potentially playing a role in the progression towards malignancy. MDM2 could be a valuable immunohistochemical staining in the head and neck pathologist’s immunohistochemical toolbox to identify the *HMGA2*-altered lesions and may explain the presence of cytonuclear atypia. However, the ‘trias’ of striking cytonuclear atypia, *HMGA2*-alteration and *MDM2* amplification is currently insufficient evidence to diagnose CXPA in the absence of the classical diagnostic criteria, and no significant association between *MDM2* status and clinical outcome was confirmed in this analysis.

## Electronic Supplementary Material

Below is the link to the electronic supplementary material.

Supplementary Material 1

Supplementary Material 2

Supplementary Material 3

## Data Availability

No datasets were generated or analysed during the current study.

## Abbreviations

PA: Pleomorphic Adenoma
CXPA: Carcinoma Ex Pleomorphic Adenoma
PLAG1: Pleomorphic Adenoma Gene 1
HMGA2: High Mobility Group AT-hook 2
MDM2: Mouse Double Minute 2
CDK4: Cyclin-Dependent Kinase 4
NGS: Next Generation Sequencing
FISH: Fluorescence In Situ Hybridization
PCR: Polymerase Chain Reaction
aCGH: array Comparative Genomic Hybridization
