# Supplementary figures and images for: Recurrent MDM2 Amplification in the Spectrum of HMGA2-Altered Pleomorphic Adenoma, Atypical Pleomorphic Adenoma and Carcinoma Ex Pleomorphic Adenoma
Source: Head Neck Pathol. 2025 May 8;19(1):56. doi: 10.1007/s12105-025-01794-y (PMC12061814; doi:10.1007/s12105-025-01794-y)

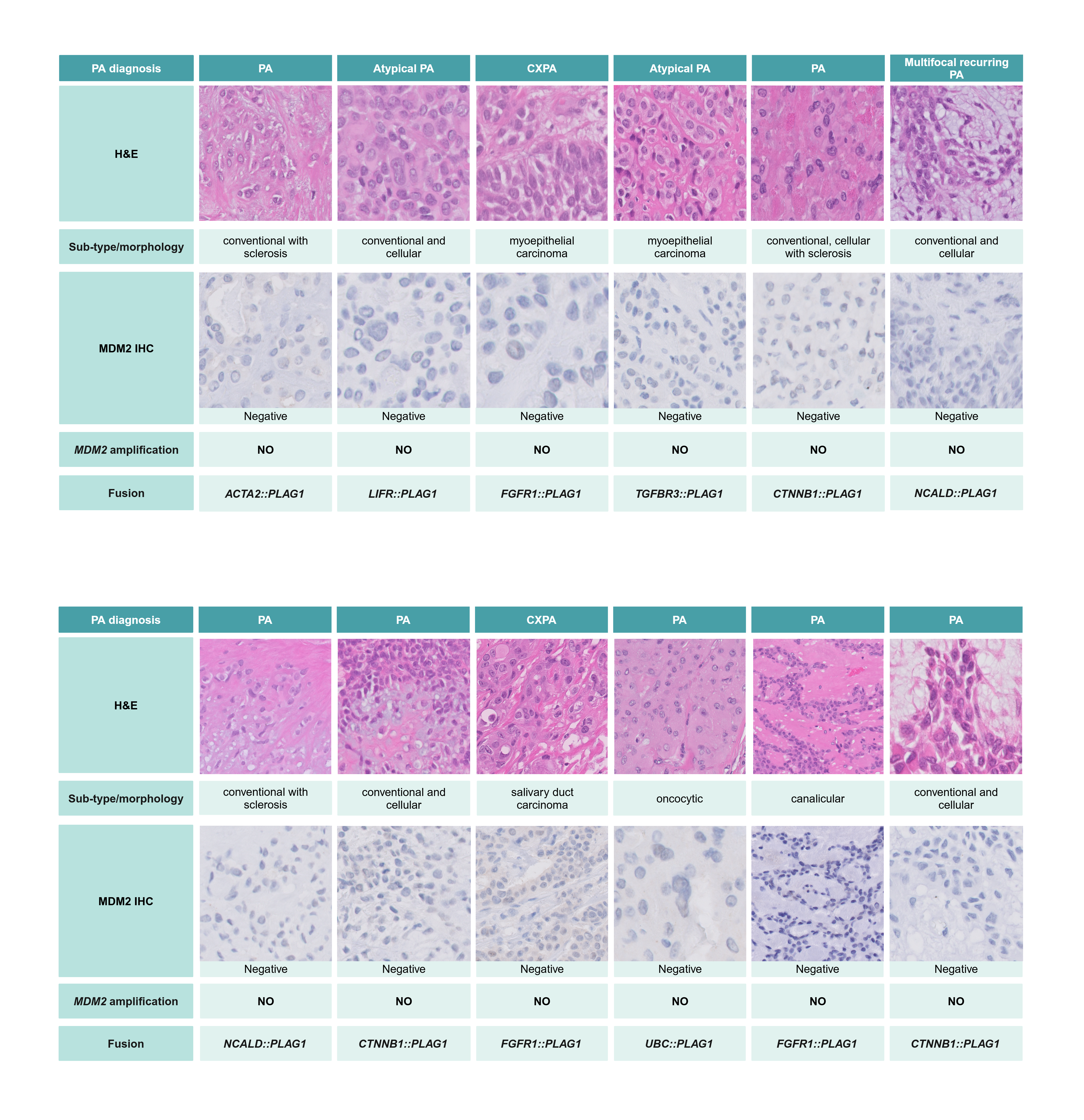

Supplement: Supplementary file 1 — Supplementary Material 1 [file 12105_2025_1794_MOESM1_ESM.tif]

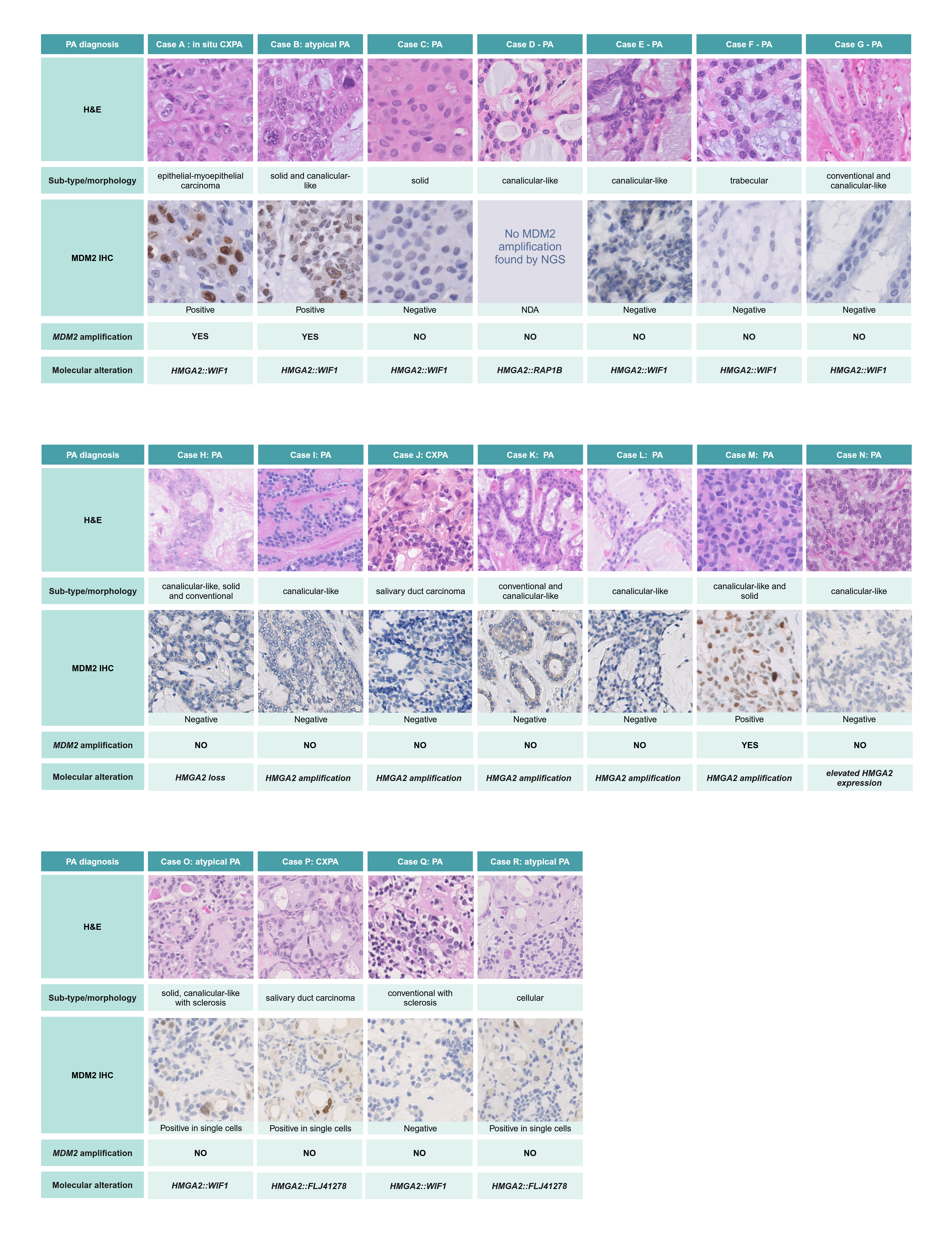

Supplement: Supplementary file 2 — Supplementary Material 2 [file 12105_2025_1794_MOESM2_ESM.tif]

**
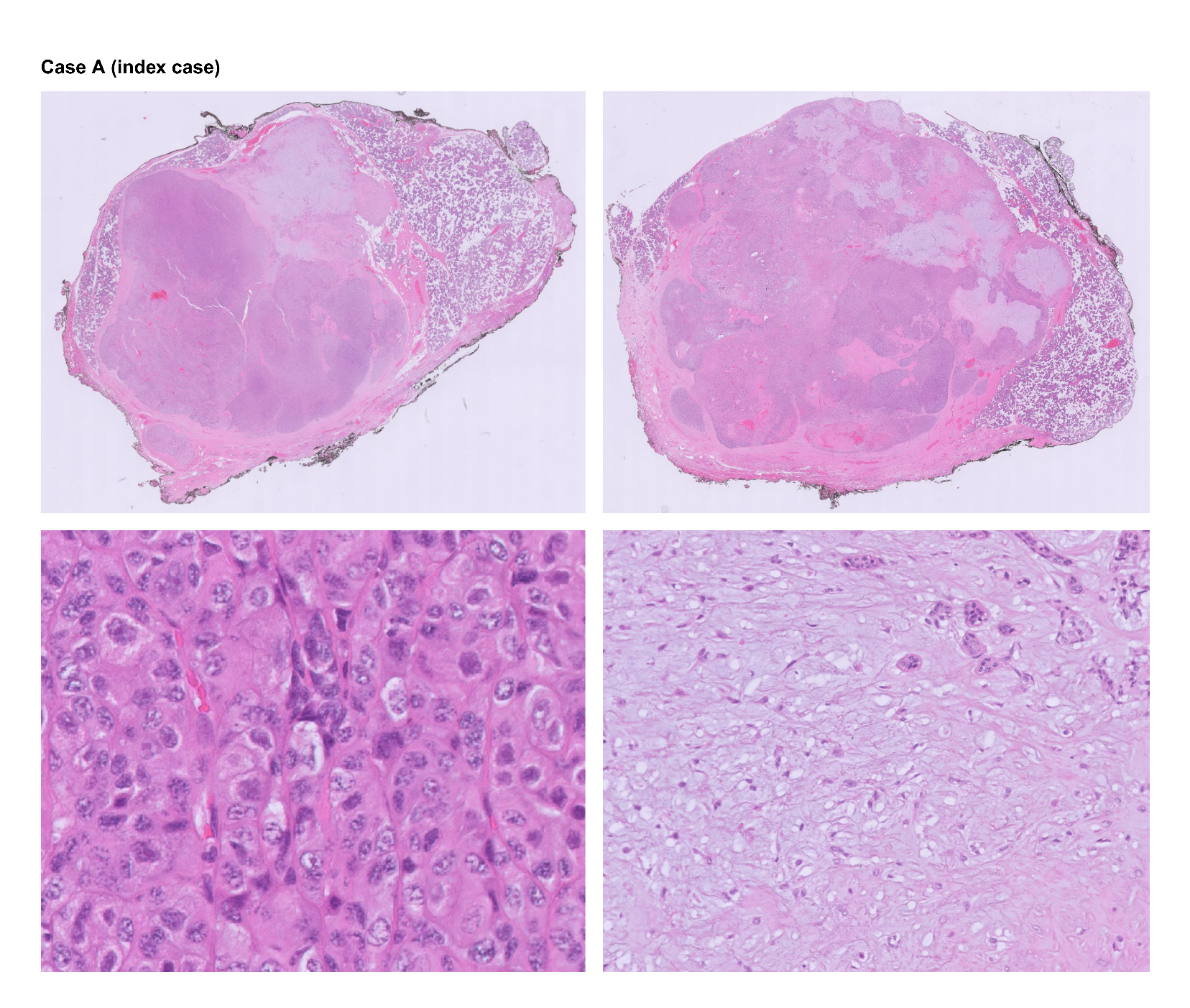
Supplementary 1 – additional histological images of 9 cases in Figure 2**

**
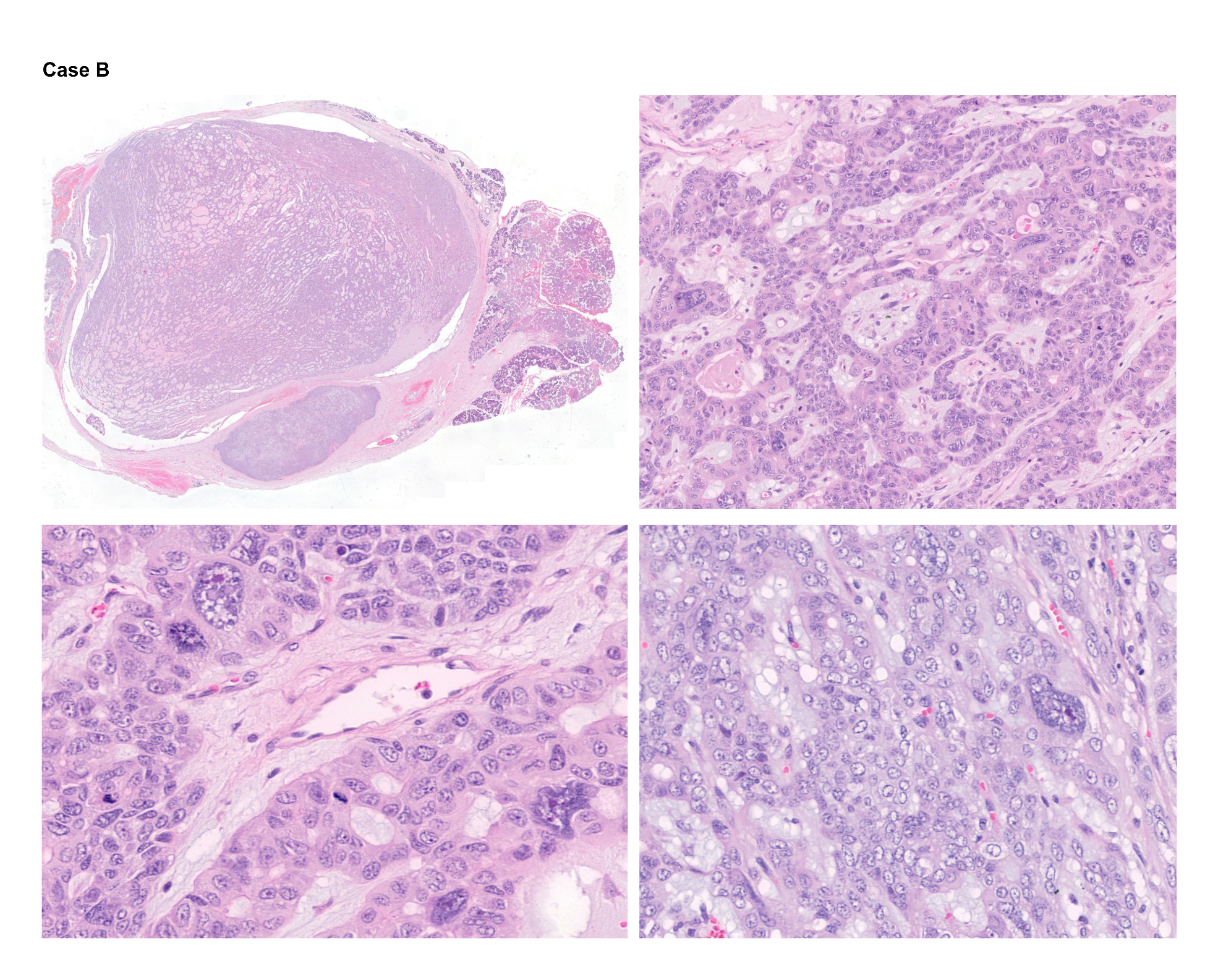
.**

**
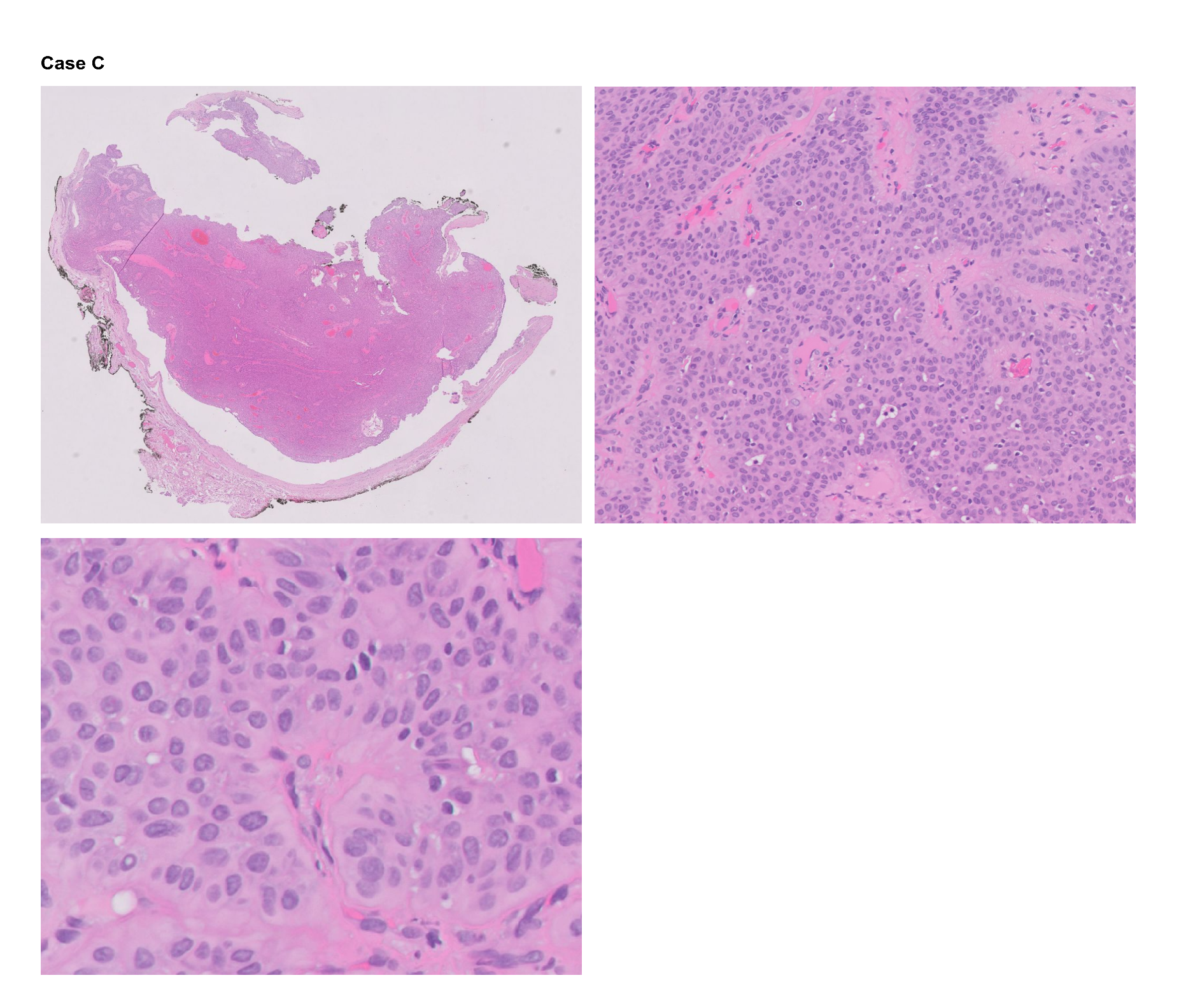
**

**
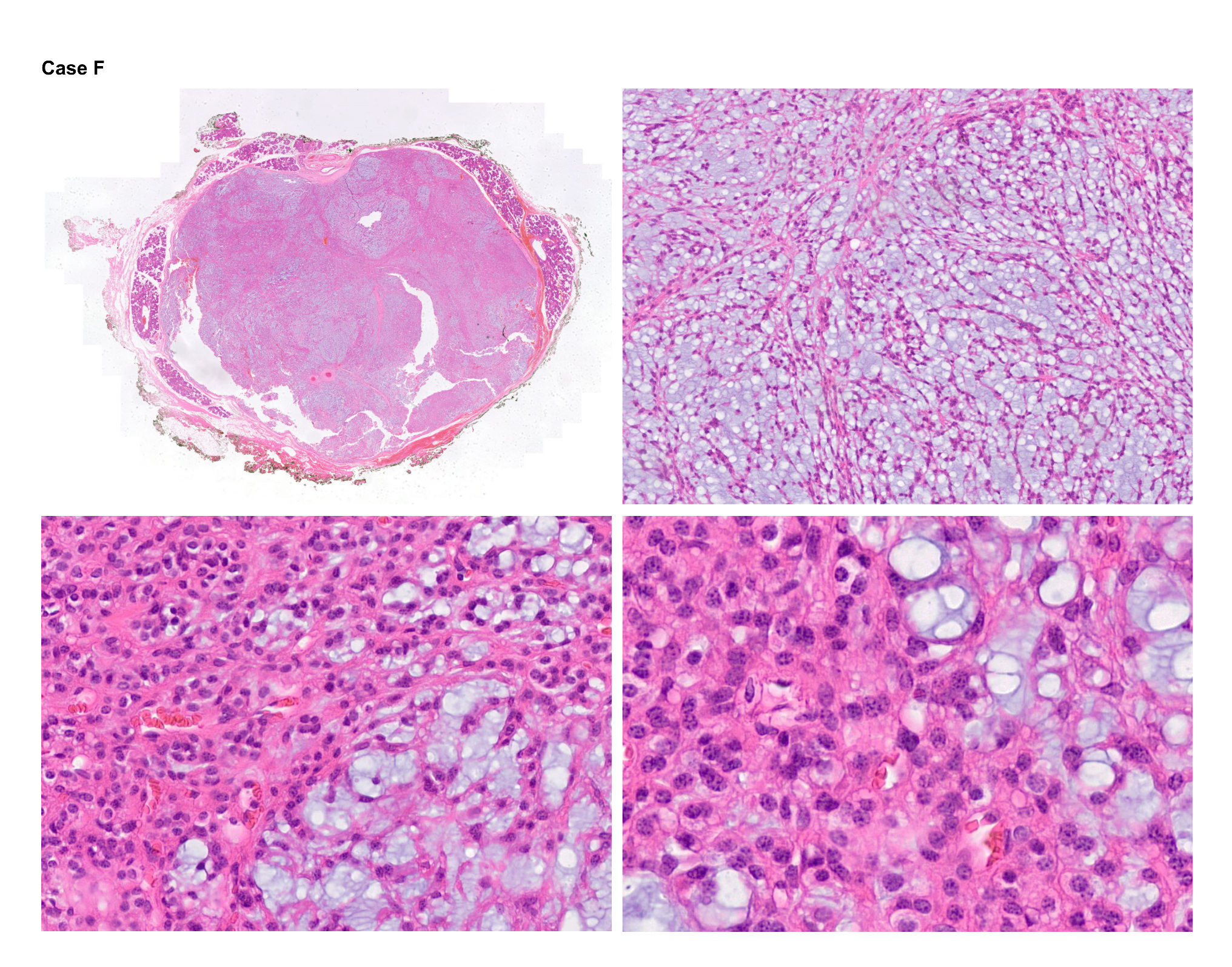
**

**
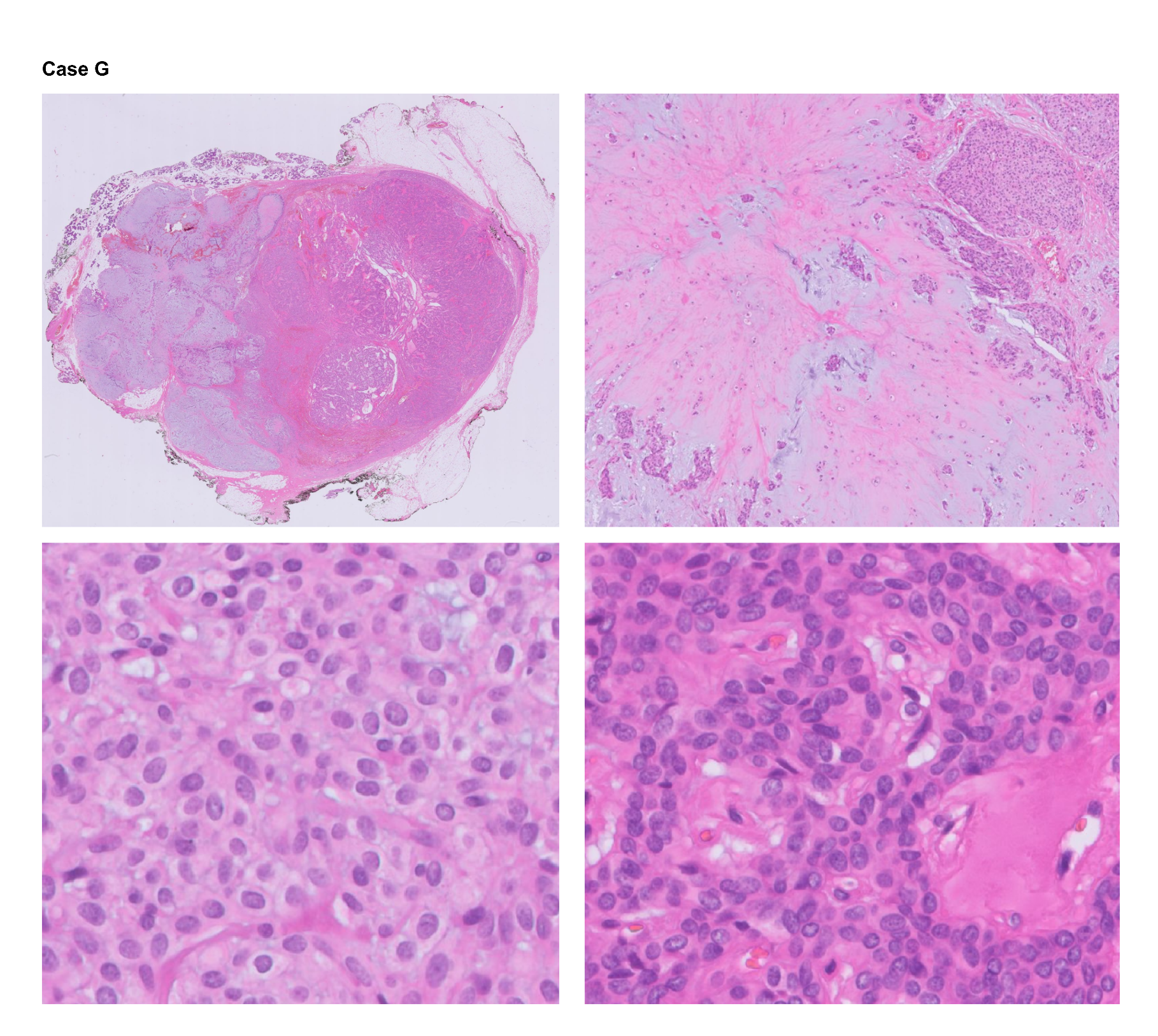
**

**
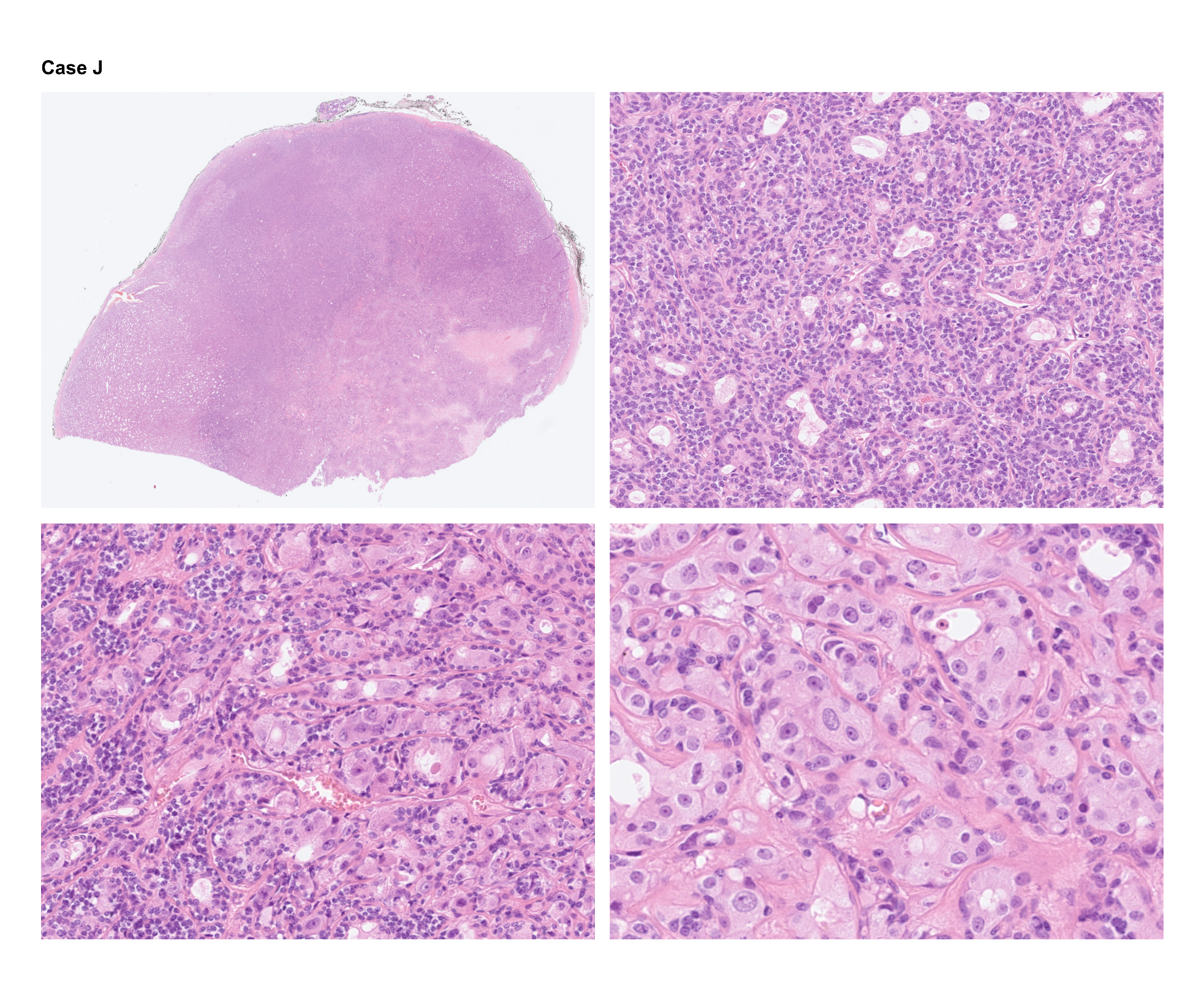
**

**
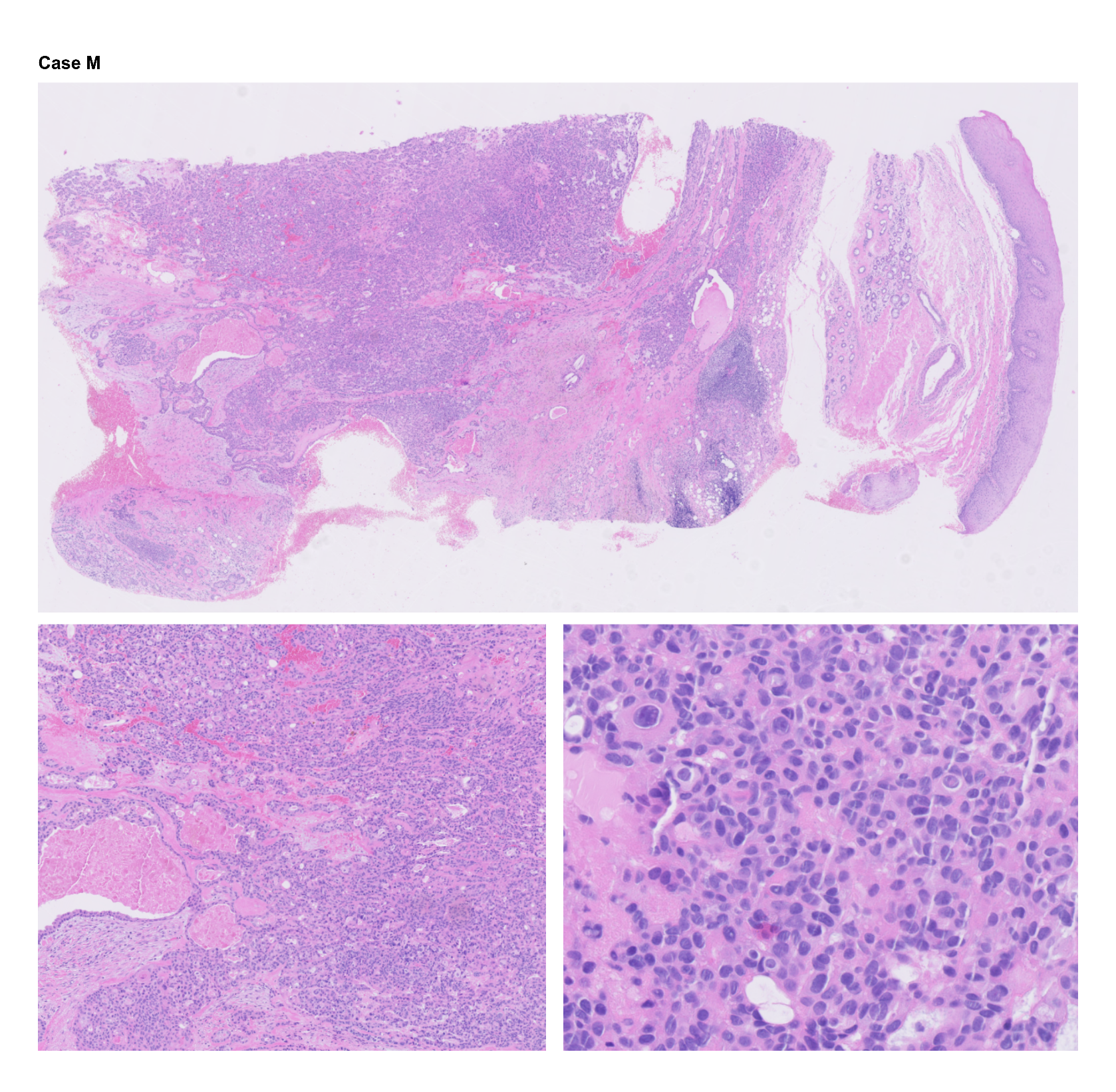
**

**
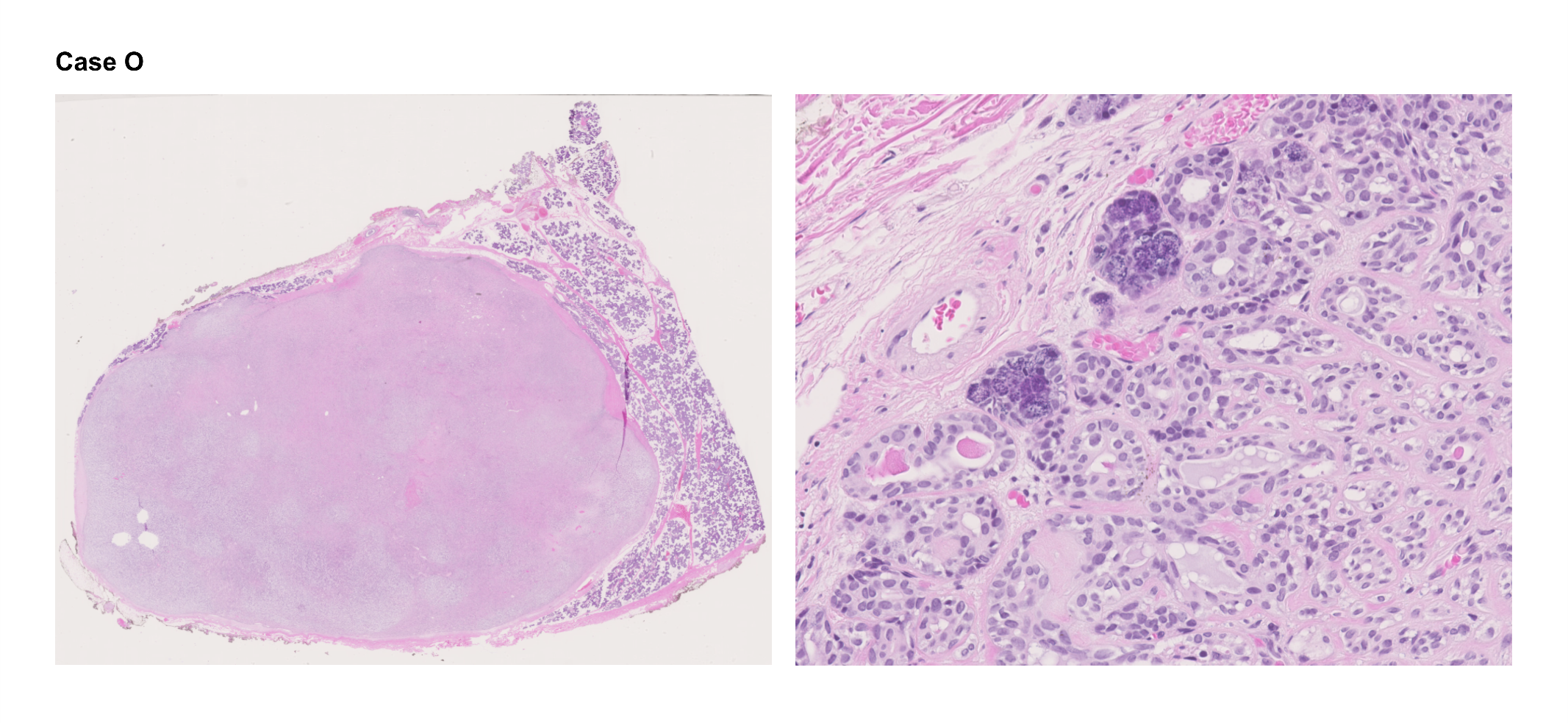
**

**
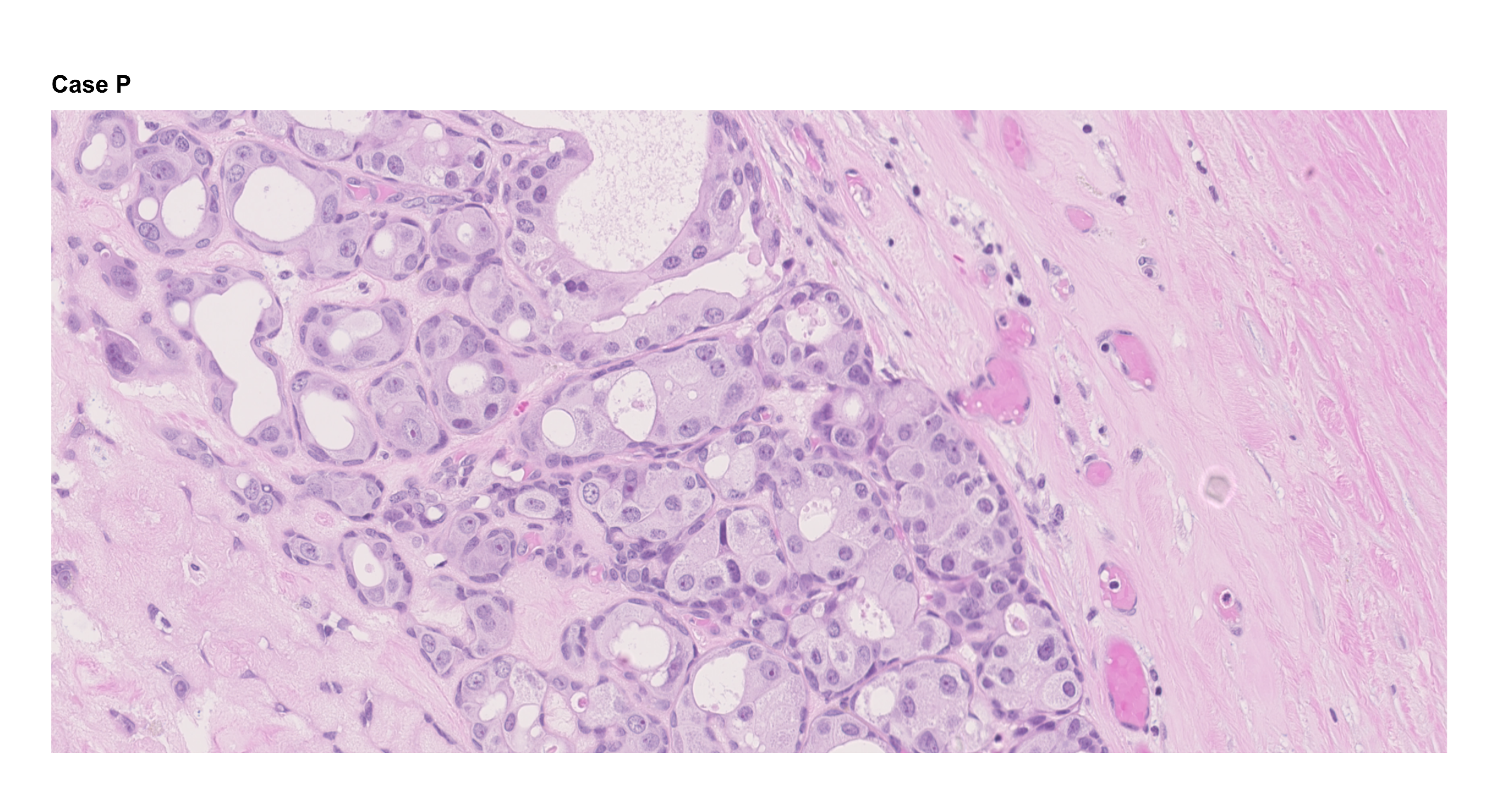
**

Supplement: Supplementary file 3 — Supplementary Material 3 [file 12105_2025_1794_MOESM3_ESM.docx]
